# Gain of power of the general regression model compared to Cochran-Armitage Trend tests: simulation study and application to bipolar disorder

**DOI:** 10.1186/s12863-017-0486-6

**Published:** 2017-03-10

**Authors:** Marie-Hélène Dizier, Florence Demenais, Flavie Mathieu

**Affiliations:** 1 Genetic Variation and Human Diseases Unit, UMR-946, Inserm, Université Paris Diderot, Université Sorbonne Paris Cité, Paris, France; 2 Inserm Siège, Université Paris Diderot, Université Sorbonne Paris Cité, Paris, France

**Keywords:** Genetic Association Studies, Simulations, GRM, Bipolar

## Abstract

**Background:**

Most genome-wide association studies assumed an additive model of inheritance which may result in significant loss of power when there is a strong departure from additivity. The General Regression Model (GRM), which allows performing an assumption-free test for association by testing for both additive effect and deviation from additive effect, may be more appropriate for association tests. Additionally, GRM allows testing the underlying genetic model. We compared the power of GRM association test to additive and other Cochran-Armitage Trend (CAT) tests through simulations and by applying GRM to a large case/control sample, the bipolar Welcome Trust Case Control Cohort data. Simulations were performed on two sets of case/control samples (1000/1000 and 2000/2000), using a large panel of genetic models. Four association tests (GRM and additive, recessive and dominant CAT tests) were applied to all replicates.

**Results:**

We showed that GRM power to detect association was similar or greater than the additive CAT test, in particular in case of recessive inheritance, with up to 67% gain in power. GRM analysis of genome-wide bipolar disorder Welcome Trust Consortium data (1998 cases/3004 controls) showed significant association in the 16p12 region (rs420259; *P* = 3.4E-7) which has not been identified using the additive CAT test. As expected, rs42025 fitted a non-additive (recessive) model.

**Conclusions:**

GRM provides increased power compared to the additive CAT test for association studies and is easily applicable.

**Electronic supplementary material:**

The online version of this article (doi:10.1186/s12863-017-0486-6) contains supplementary material, which is available to authorized users.

## Background

During the last decades, numerous genetic association studies for diseases or traits have been applied to large panels of SNPs (for single-nucleotide polymorphism), either at the genome-wide level (Genome Wide Association Studies (GWAS)) or in candidate regions. To limit the multiple testing problem, association studies were usually based on a single association test statistic between each SNP and disease. However, it is not obvious which test should be used. The simplest association test is allele-based and requires the strong assumption of Hardy Weinberg (HW) equilibrium. Model-based tests such as the Cochran-Armitage Trend (CAT) tests [1] have the advantage of not requiring this assumption and have thus been recommended for association studies [2]. CAT tests have been designed for different genetic models of the SNP effect on disease: additive (CAT_ADD), dominant (CAT_DOM) and recessive (CAT_REC), depending on the coding scheme assigned to the three genotypes. As the true genetic model is often unknown, CAT_ADD test is commonly used as it can represent an intermediate test between recessive and dominant tests. A major disadvantage of CAT tests is their sensitivity to model misspecification, as they are model-based. In case of deviation from additivity, the power of CAT_ADD test to detect association may be decreased [3–5]. A new likelihood-based method, which compares allelic frequencies between cases and controls and does not require specification of the genetic model or HW equilibrium assumption has recently been proposed. However the power of this approach did not exceed that of the CAT_ADD test [6].

Other tests, such as the “maximin efficiency robusts tests” (MERT and MAX), which are based on efficiency robustness theory, have relatively high power for any of the three commonly used genetic models (additive, recessive and dominant) [4]. The MERT test is a linear combination of the standardized optimal tests (additive, recessive and dominant) while the MAX test is the maximum of the standardized optimal tests. However, these tests are computationally intensive.

When the underlying genetic model is unknown, the General Regression Model (GRM), which includes both a term for additive effect and a term for deviation from additivity (dominance term) may be more appropriate for association tests. The GRM allows to first testing for association without making assumption on the mode of transmission and then testing for the underlying genetic model. The goal of this study was to compare the power of the GRM test for association with those of the most commonly used CAT_ADD test as well as CAT_DOM and CAT_REC tests through a simulation study that considered a large panel of genetic models. We then applied GRM and CAT_ADD tests to the bipolar disorder Wellcome Trust Cases-Controls Cohort’ data (WTCCC), in order to assess whether GRM was able to replicate CAT_ADD test results and to detect additional loci.

## Methods

### Association tests

#### CAT tests

The CAT tests can be applied to different genetic models. They are based on a logistic regressive model such that: logit(P) = α + β (X), where X is equal to 0, 1 and 2 for each of three SNP genotypes (AA, Aa, aa respectively) in case of an additive model (CAT_ADD); 0, 0, 1 for a recessive model (CAT_REC) and 0, 1, 1, for a dominant model (CAT_DOM) (see Table 1 for details on the coding scheme). The association test (β = 0 under H0) is a likelihood-ratio test which asymptotically follows a Chi-square distribution with one degree of freedom (df).

**Table 1:** Coding scheme of each genotype used for each CAT model and GRM

	CAT models			GRM	
Genotypes	Additive (CAT_ADD)	Dominant (CAT_DOM)	Recessive (CAT_REC)	Add	DomDev
AA	0	0	0	0	0
Aa	1	1	0	1	1
aa	2	1	1	2	0

#### General regression model

The General Regression Model (GRM), which includes two terms, an additive term and a dominance term (deviation from additivity), as proposed by Fisher and Wilson [7], allows testing for association without making assumption on the genetic model. The logistic regression model is written as:

$$ \mathrm{logit}\left(\mathrm{P}\right)=\upalpha +{\upbeta}_{\mathrm{Add}}\left(\mathrm{Add}\right)+{\upbeta}_{\mathrm{DomDev}}\left(\mathrm{Domdev}\right) $$

where β_Add_ is the regression coefficient for the additive effect (coded as 0, 1, 2 for the three genotypes AA, Aa, aa, see Table 1) and β_DomDev_ is the regression coefficient for the dominance term (coded as 0, 1, 0, see Table 1). The test for association (β_Add_ = β_DomDev_ = 0 under H0) is a likelihood-ration test which is assumed to follow a chi-square distribution with 2 df.

**Fig. 1:**
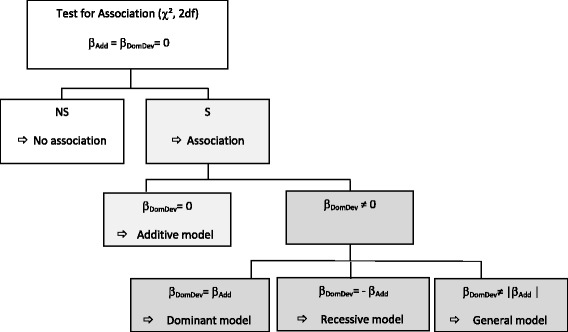
Statistical decisional diagram to test the genetic model using GRM. S and NS: significant and non-significant respectively

If there is significant evidence for association, the following genetic models can then be examined: by setting β_DomDev_ = 0 for the additive model, β_DomDev_ = β_Add_ for the dominant model β_DomDev_ = - β_Add_ for the recessive model. The decision tree is shown in Fig. 1.

The underlying genetic model is only tested if the association test is significant. First the additive model (Under H0, β_DomDev_ = 0) is tested. If H0 is not rejected, the additive model is retained. If the additive model is rejected, the dominant and recessive models are then tested: 1/if (β_DomDev_ = β_Add_) is not rejected, the dominant model is retained and 2/if (β_DomDev_ = -β_Add_) is not rejected, the recessive model is retained (see Fig. 1).

### Simulation studies

A total of 200 000 or 1.0E8 replicates (for power or type 1 error estimation respectively) of samples of 1000 cases and 1000 controls were simulated. A binary trait was generated, using three different prevalence of disease (1%, 5 and 10%). We considered three genetic models (additive, dominant and recessive) for the causal variant. For each of these models, the minor allele frequency (MAF) was set at 0.1, 0.2, 0.3 or 0.4, and, for each MAF, the Odds-Ratios (OR) were varied between 1.0 and 3.2 (with a step of 0.2). Association analyses were performed for all simulated replicates using GRM and the CAT tests (CAT_ADD, CAT_DOM and CAT_REC). Thresholds of 1.0E-5 and 1.0E-7 were used to declare significance as currently used in association studies of large panels of markers.

### Type one error rate

To estimate the type one error rate, simulations were done under the null hypothesis of no association (OR = 1.0 under H0). The type one error rate was estimated by the proportion of replicates showing significant association using either GRM or the CAT tests, for three significance thresholds: 5, 1% and 1.0E-5.

### Comparison of power of association tests

Empirical power of each statistical test was estimated by the proportion of simulated replicates showing significant association.

### Test of genetic model

For each simulated model (additive, dominant or recessive), the proportion of replicates retaining the true model was estimated among all replicates showing significant association.

### Sample size effect

To assess the sensitivity of our results to sample size, samples of 2000 cases and 2000 controls were also generated for all genetic models and combinations of parameter values (MAF, ORs).

## Results

### Type one error rate

Under the null hypothesis of no association, the estimated type I error rate was equal or close to the three theoretical thresholds considered of 5, 1% and 1.0E-5. Results are provided in Table 2.

**Table 2:** Type one error rate

Theoretical thresholds	Type one error			
	CAT_ADD	CAT_REC	CAT_DOM	GRM
5%	5.0%	5.0%	5.0%	4.8%
1%	1.0%	1.0%	1.0%	0.9%
1.0E-5	1.0E-5	0.7E-5	1.0E-5	0.6E-5

### Comparison of power of association tests

**Fig. 2:**
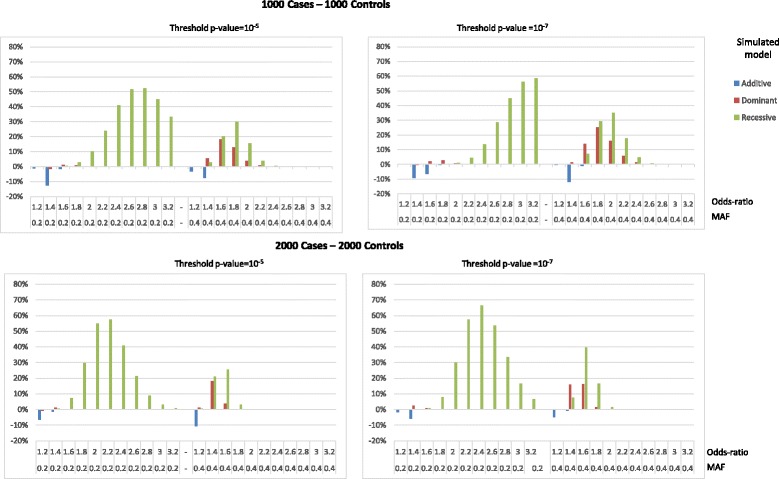
Differences of power between GRM and CAT_ADD tests to detect association depending on Odds-ratio and minor allele frequency

Results were similar for the three disease prevalence (1%, 5 and 10%). For sake of simplicity, only results obtained for a prevalence of 5% are provided. Results for simulated samples of 1000 cases/1000 controls are shown in Fig. 2 for MAFs of 0.2 and 0.4 and in Tables 3 and 4 for all MAFs.

**Table 3:** Power of GRM and CAT tests to detect association for a P-value threshold of 1.0E-5 using a sample size of 1000 cases/1000 controls

		Simulated model: Additive				Simulared model: Dominant				Simulated model: Recessive			
		Tests :				Tests :				Tests :			
MAF	OR	CAT_DOM	CAT_REC	CAT_ADD	GRM	CAT_DOM	CAT_REC	CAT_ADD	GRM	CAT_DOM	CAT_REC	CAT_ADD	GRM
0.1	1.2	0	0	0.01	0	0	0	0	0	0	0	0	0
0.1	1.4	0.17	0	0.20	0.11	0.12	0	0.11	0.07	0	0	0	0
0.1	1.6	0.71	0.01	0.77	0.64	0.59	0	0.54	0.47	0	0	0	0
0.1	1.8	0.97	0.06	0.99	0.96	0.93	0	0.91	0.88	0	0	0	0
0.1	2	1	0.23	1	1	1	0	0.99	0.99	0	0	0	0
0.1	2.2	1	0.51	1	1	1	0	1	1	0	0.01	0	0
0.1	2.4	1	0.78	1	1	1	0	1	1	0	0.01	0	0
0.1	2.6	1	0.93	1	1	1	0	1	1	0	0.03	0	0
0.1	2.8	1	0.99	1	1	1	0	1	1	0	0.06	0	0
0.1	3	1	1	1	1	1	0.01	1	1	0	0.10	0	0
0.1	3.2	1	1	1	1	1	0.01	1	1	0	0.16	0	0
0.2	1.2	0.02	0	0.03	0.01	0.01	0	0.01	0	0	0	0	0
0.2	1.4	0.49	0.05	0.61	0.48	0.29	0	0.22	0.20	0	0	0	0
0.2	1.6	0.96	0.39	0.99	0.97	0.85	0	0.75	0.76	0	0.02	0	0.01
0.2	1.8	1	0.83	1	1	0.99	0	0.98	0.98	0	0.08	0.01	0.03
0.2	2	1	0.98	1	1	1	0.01	1	1	0	0.24	0.02	0.12
0.2	2.2	1	1	1	1	1	0.02	1	1	0	0.46	0.05	0.29
0.2	2.4	1	1	1	1	1	0.03	1	1	0	0.70	0.12	0.53
0.2	2.6	1	1	1	1	1	0.04	1	1	0.01	0.86	0.22	0.74
0.2	2.8	1	1	1	1	1	0.06	1	1	0.01	0.95	0.36	0.88
0.2	3	1	1	1	1	1	0.08	1	1	0.02	0.98	0.51	0.96
0.2	3.2	1	1	1	1	1	0.11	1	1	0.03	1	0.65	0.99
0.3	1.2	0.03	0.01	0.06	0.03	0.01	0	0.01	0.01	0	0	0	0
0.3	1.4	0.61	0.25	0.81	0.71	0.31	0	0.19	0.22	0	0.02	0	0.01
0.3	1.6	0.98	0.83	1	1	0.85	0	0.68	0.77	0	0.17	0.03	0.10
0.3	1.8	1	0.99	1	1	0.99	0.01	0.95	0.98	0	0.51	0.12	0.38
0.3	2	1	1	1	1	1	0.01	1	1	0	0.83	0.32	0.73
0.3	2.2	1	1	1	1	1	0.03	1	1	0.02	0.97	0.57	0.93
0.3	2.4	1	1	1	1	1	0.04	1	1	0.03	1	0.80	0.99
0.3	2.6	1	1	1	1	1	0.07	1	1	0.07	1	0.93	1
0.3	2.8	1	1	1	1	1	0.09	1	1	0.12	1	0.98	1
0.3	3	1	1	1	1	1	0.12	1	1	0.19	1	1	1
0.3	3.2	1	1	1	1	1	0.15	1	1	0.28	1	1	1
0.4	1.2	0.03	0.02	0.08	0.04	0.01	0	0	0	0	0	0	0
0.4	1.4	0.61	0.47	0.87	0.80	0.23	0	0.10	0.15	0	0.09	0.02	0.05
0.4	1.6	0.98	0.96	1	1	0.75	0	0.46	0.64	0	0.48	0.16	0.36
0.4	1.8	1	1	1	1	0.97	0.01	0.81	0.94	0.01	0.88	0.50	0.80
0.4	2	1	1	1	1	1	0.01	0.96	1	0.03	0.99	0.82	0.97
0.4	2.2	1	1	1	1	1	0.02	0.99	1	0.06	1	0.96	1
0.4	2.4	1	1	1	1	1	0.03	1	1	0.13	1	1	1
0.4	2.6	1	1	1	1	1	0.05	1	1	0.23	1	1	1
0.4	2.8	1	1	1	1	1	0.07	1	1	0.36	1	1	1
0.4	3	1	1	1	1	1	0.09	1	1	0.50	1	1	1
0.4	3.2	1	1	1	1	1	0.11	1	1	0.64	1	1	1

**Table 4:** Power of GRM and CAT tests to detect association for a P-value threshold of 1.0E-7 using a sample size of 1000 cases/1000 controls

		Simulated model: Additive				Simulated model: Dominant				Simulated model: Recessive			
		Tests:				Tests:				Tests:			
MAF	OR	CAT_DOM	CAT_REC	CAT_ADD	GRM	CAT_DOM	CAT_REC	CAT_ADD	GRM	CAT_DOM	CAT_REC	CAT_ADD	GRM
0.1	1.2	0	0	0	0.00	0	0	0	0	0	0	0	0
0.1	1.4	0.03	0	0.04	0.02	0.02	0	0.02	0.01	0	0	0	0
0.1	1.6	0.36	0	0.42	0.27	0.25	0	0.21	0.16	0	0	0	0
0.1	1.8	0.85	0	0.89	0.80	0.72	0	0.66	0.60	0	0	0	0
0.1	2	0.99	0.03	1	0.99	0.96	0	0.94	0.92	0	0	0	0
0.1	2.2	1	0.14	1	1	1	0	0.99	0.99	0	0	0	0
0.1	2.4	1	0.38	1	1	1	0	1	1	0	0	0	0
0.1	2.6	1	0.67	1	1	1	0	1	1	0	0	0	0
0.1	2.8	1	0.88	1	1	1	0	1	1	0	0	0	0
0.1	3	0.99	0.96	1	1	1	0	1	1	0	0.01	0	0
0.1	3.2	0.99	0.99	1	1	1	0	1	1	0	0.02	0	0
0.2	1.2	0	0	0	0	0	0	0	0	0	0	0	0
0.2	1.4	0.17	0	0.26	0.17	0.07	0	0.04	0.04	0	0	0	0
0.2	1.6	0.81	0.10	0.91	0.84	0.54	0	0.41	0.43	0	0	0	0
0.2	1.8	0.99	0.50	1	1	0.93	0	0.85	0.88	0	0.01	0	0
0.2	2	1	0.88	1	1	1	0	0.99	0.99	0	0.05	0	0.01
0.2	2.2	1	0.99	1	1	1	0	1	1	0	0.14	0.01	0.05
0.2	2.4	1	1	1	1	1	0	1	1	0	0.33	0.02	0.15
0.2	2.6	1	1	1	1	1	0	1	1	0	0.55	0.04	0.33
0.2	2.8	1	1	1	1	1	0.01	1	1	0	0.75	0.10	0.54
0.2	3	1	1	1	1	1	0.01	1	1	0	0.89	0.18	0.74
0.2	3.2	1	1	1	1	1	0.01	1	1	0	0.96	0.29	0.88
0.3	1.2	0	0	0.01	0	0	0	0	0	0	0	0	0
0.3	1.4	0.27	0.05	0.48	0.36	0.08	0	0.04	0.05	0	0	0	0
0.3	1.6	0.90	0.52	0.98	0.96	0.55	0	0.33	0.44	0	0.03	0	0.01
0.3	1.8	1	0.94	1	1	0.93	0	0.77	0.88	0	0.18	0.02	0.10
0.3	2	1	1	1	1	1	0	0.96	0.99	0	0.51	0.08	0.36
0.3	2.2	1	1	1	1	1	0	1	1	0	0.81	0.23	0.69
0.3	2.4	1	1	1	1	1	0	1	1	0	0.96	0.46	0.91
0.3	2.6	1	1	1	1	1	0.01	1	1	0.01	0.99	0.70	0.98
0.3	2.8	1	1	1	1	1	0.01	1	1	0.02	1	0.87	1
0.3	3	1	1	1	1	1	0.02	1	1	0.04	1	0.95	1
0.3	3.2	1	1	1	1	1	0.03	1	1	0.07	1	0.99	1
0.4	1.2	0	0	0.01	0	0	0	0	0	0	0	0	0
0.4	1.4	0.26	0.16	0.58	0.47	0.05	0	0.01	0.03	0	0.01	0	0
0.4	1.6	0.88	0.80	0.99	0.98	0.40	0	0.16	0.30	0	0.17	0.03	0.10
0.4	1.8	1	0.99	1	1	0.83	0	0.49	0.74	0	0.59	0.18	0.47
0.4	2	1	1	1	1	0.98	0	0.79	0.95	0	0.91	0.50	0.85
0.4	2.2	1	1	1	1	1	0	0.94	0.99	0.01	0.99	0.80	0.98
0.4	2.4	1	1	1	1	1	0	0.99	1	0.02	1	0.95	1
0.4	2.6	1	1	1	1	1	0.01	1	1	0.05	1	0.99	1
0.4	2.8	1	1	1	1	1	0.01	1	1	0.10	1	1	1
0.4	3	1	1	1	1	1	0.01	1	1	0.18	1	1	1

When the simulated model was additive, the power of GRM and CAT_ADD tests to detect association were similar, for both critical thresholds of 1.0E-5 and 1.0E-7. For ORs less than or equal to 1.8, the CAT_ADD was slightly more powerful than GRM only for a few situations, with an increase in power never exceeding 15%, for all MAFs and P-value thresholds. For highest ORs, there was no difference as all power estimates reached 1.

When the simulated model was dominant, the GRM test was as powerful as the CAT_ADD test for a MAF of 0.2. For a MAF of 0.4, GRM was slightly more powerful, with highest gains in power reaching 18% for OR = 1.6 and significance threshold of 1.0E-5 or 25% for OR = 1.8 and threshold of 1.0E-7. As expected the CAT_DOM test had always the highest power when the simulated model was dominant, but the difference with the GRM never exceeded 12%.

When the simulated model was recessive, the GRM test was always more powerful than the CAT_ADD test, especially for SNP allele frequency of 0.2, with a gain in power of 52% (for OR = 2.6 and P =1.0E-5) or 59% (for OR = 3.2 and P =1.0E-7). When the MAF was 0.4, the gains in power were smaller but were obtained for lower ORs (30% for OR = 1.8 and P =1.0E-5 or 35% for OR = 2 and P =1.0E-7). As expected, the CAT_REC test also had the highest power when the simulated model was recessive, but the difference in power with respect to GRM never exceeded 22%. For ORs less than 1.4, there was no difference as all power estimates were close to 0 for all tests.

Using a larger sample size of 2000 cases/2000 controls (results provided in Fig. 2, Additional file 1: Table S1 and S2), similar conclusions could be drawn for the power comparison between GRM and CAT_ADD tests, for all simulated model. However, the strongest gain in power of GRM test versus CAT_ADD test increased and was obtained for smaller ORs. For example, for a MAF of 0.2 the highest gain in power with a recessive simulated model reached 67% (OR = 2.4 and P =1.0E-7) and, when the MAF was 0.4, the power gain reached 40% (OR = 1.6 and P =1.0E-7).

### Tests of genetic model

**Fig. 3:**
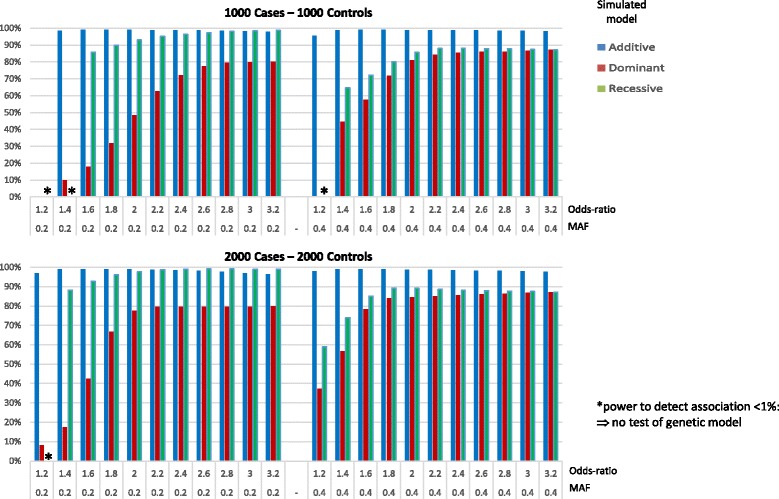
Proportion of replicates retaining the true model at *P* = 1%, among replicated showing significant association (*P* = 1.0E-5)

Results for both simulated sample sizes are provided in Fig. 3. The genetic model was tested only for SNP(s) significantly associated with the disease at the critical threshold of 1.0E-5. The test of the genetic model was based on a less stringent threshold of 0.01, as it only applies to SNP(s) showing significant association. When the power to detect association was less than 1%, tests of genetic models were not performed to avoid a bias in the estimation of the true model detection. For a sample of 1000 cases/1000 controls, when data were simulated under an additive model, the true model was retained in most replicates. As expected, the proportion of replicates retaining the true model was close to [1 - type 1 error] ranging between 98 and 99%.

When data were simulated under a dominant model, the true model was retained in most replicates; for an OR greater than 2, the proportion of replicates retaining the true model ranged between 62 and 87%. For an OR less than or equal to 2, this proportion was smaller and depended on the MAF: ranging between 10 and 48% for a MAF of 0.2 and between 45 and 81% for a MAF of 0.4.

When data were simulated under a recessive model, the true model was retained by GRM in more than 70% of replicates (ranging between 72 and 99%) for an OR greater than or equal to 1.6, for all MAFs.

When the data were generated in a larger sample size of 2000 cases/2000 controls, the proportion of replicates retaining the true model was increased for all simulated models (see Fig. 3).

We can notice that not concluding to the true model, when it was dominant or recessive, was mostly due to lack of power to reject an additive model (β_DomDev_ = 0, see Additional file 2: Figure S1). This lack of power was observed for smallest ORs and decreased when the sample size increased.

### Application to the WTCCC Bipolar data

#### Sample description

We obtained approval for using the raw genotype and phenotypic data for the original WTCCC bipolar disorder (BD) data set. The dataset consisted of 1998 BD cases and 3004 controls genotyped using the Affymetrix 500K array (see WTCCC 2007 [8] for details). We applied similar quality control (QC) filtering as the original WTCCC 2007 study, i.e. 1) individual samples excluded in case of missing data across all SNPs >3% or genome-wide heterozygosity greater than 30% or lower than 23%, 2) SNPs excluded in case of MAF < 5% or significant deviation from HW equilibrium in controls (P <5.7E-7) or between the two controls groups (P <5.7E-7). A total of 371 137 SNPs were retained for analysis.

### Test of association

For a critical threshold of 5.0E-7 (as used in the original WTCCC 2007) the GRM test showed significant association of BD with one SNP located in the 16p12 region: rs420259 (*P* =3.4E-7) (see Table 5 for details), whereas the CAT_ADD test did not (*P* =9.3E-4). Note that no other SNP was detected by either GRM or CAT_ADD test.

**Table 5:** Results of GRM association test in bipolar disorder WTCCC case-control sample (WTCCC 20007)

A: Test for association								
Chromosome	SNP id		Rsid		Position		GRM *P*-value	CAT_ADD *P*-value
2	SNP_A-1964333		rs7570682		104349699		4,26E-6	7,91E-7
2	SNP_A-1916900		rs11123306		115948251		4,77E-6	7,53E-7
2	SNP_A-2300074		rs1375144		115957416		8,18E-6	1,25E-6
3	SNP_A-2266670		rs4276227		32305690		2,16E-5	3,47E-6
6	SNP_A-4217035		rs6458307		42839093		3,38E-6	0,28
9	SNP_A-2106829		rs10982256		114340388		3,73E-5	6,59E-6
14	SNP_A-2284698		rs10134944		57188949		2,67E-6	1,91E-6
14	SNP_A-4304670		rs11622475		103578829		1,11E-5	2,17E-6
**16**	**SNP_A-2248415**		**rs420259**		**23541527**		**3,37E-7**	**9.3E-4**
16	SNP_A-2306762		rs1344484		51469800		6,81E-6	1,03E-6
20	SNP_A-1909934		rs3761218		3724175		9,96E-6	2,24E-5
B Test for genetic model								
SNP id	rsid	β__ADD_	IC_β_ADD_ (99%)		β__ADD_ *P*-value	β__DomDev_	β__DomDev_ *P*-value	Genetic Model
SNP_A-2248415	rs420259	−0.33	−0.49	1.32	1.57E-7	0.35	5.56E-6	Recessive

In bold: *p* < 5.0E-7

Using a less stringent threshold (5.0E-5) to detect “suggestive” association, 10 SNPs (in addition to rs420259) were detected by GRM test. Results are detailed in Table 5a. Among them, 9 SNPs were detected by both CAT_ADD and GRM tests and 1 SNP was detected only by the GRM test.

### Test of genetic model

For the SNP rs420259 significantly associated to BD using GRM, the additive model was rejected (P =1.6E-7) and the recessive model was retained (i.e. β_ADD_ = -β_DomDev_ was not rejected). A lower risk was observed for the risk allele homozygote carriers, with an Odds-ratio of 0.75 IC (95%) = [0.67 - 0.84]) (see Table 5b for details).

## Discussion

Genetic association studies are usually conducted using the CAT_ADD test which is model based and known to be sensitive to model misspecification. Indeed, when there is departure from additivity, this test may lead to decrease in power to detect association [3–5].

Our simulation study showed that the GRM test, which does not make any assumption on the genetic model, is as powerful as or even more powerful to detect association than the CAT_ADD test. An important finding is that GRM and CAT_ADD tests had similar power when the true model was additive. In the latter situation, the decrease in power never exceeded 15%, although the GRM test has an additional degree of freedom as compared to the CAT_ADD test. We also showed that the GRM association test may be more powerful than the CAT_ADD test when the true model was dominant and even more when it was recessive. The gain in power reached 67% for a recessive model when using a significance threshold of 1.0E-7, as currently done in GWAS. This increase in power was higher for increased sample size, especially for low ORs. Thus, the advantage of GRM test over CAT_ADD test will be particularly important for multifactorial diseases where most associated variants have small ORs and which require large sample sizes to detect association.

The two maximin efficiency robust tests which were developed by Freidlin et al [4] to have relatively high power for any of the three additive, dominant and recessive models, are computationally very intensive because of permutation testing. The MAX test which is generally more powerful but even more computationally intensive than MERT [4], has been extended to derive the exact and/or the asymptotic distribution of the test statistic to be less computationally intensive [9]. Note however that this test remains twice as computationally intensive as the logistic regression-based test [10]. Moreover, MAX test is very sensitive to allele frequency: for a frequency lower than 0.3, it has smallest power than CAT_ADD under dominant and additive models [10] whereas GRM has similar power as CAT_ADD. Under other models, MAX test is always less powerful than the genotypic test [10] and consequently than the GRM test, as the genotypic and GRM tests have similar power, as expected (personal data). Based on these findings, we can argue that the power of the MAX test never exceeds the power of the GRM test. Moreover, a power comparison between MAX and GRM tests for a few number of models showed similar or higher power of GRM comparing to MAX (results not shown).

A major advantage of the GRM test is that it allows to test the underlying genetic model in the same modelling framework, whereas the genotypic test, CATs and the MAX tests do not. GRM might also be further developed to estimate and test more complex models, as it has already been done in case of gene x gene interaction [11]. GRM can be applied to association studies of large panels of markers but can also be used to perform gene-based or pathway-based analyses.

Re-analysis of WTCCC cases-controls bipolar disorder data illustrates the gain in power of GRM association test as compared to CAT_ADD test, especially when there is deviation from additivity. Using the classical GWAS threshold of 5.0E-7, the GRM test detected one SNP, significantly associated with BP, whereas CAT_ADD test did not. As expected, deviation from additivity was observed for this SNP and the recessive model was retained.

Ten additional SNPs showed suggestive association at the threshold of 5.0E-5, 9 of these SNPs were detected by both GRM and CAT_ADD tests and one SNP was detected by GRM test only. This shows once again that GRM can not only replicate results of CAT_ADD test but also allows detecting additional SNPs.

Association of BD with the rs420259 SNP, as found here using GRM test, has been initially reported by the Welcome Trust Consortium by applying the genotypic test [8], which represents a general modeling framework as GRM and genotypic tests has similar power. Interestingly, association of the same SNP with BD was also reported by applying either the MAX test [12] or a score-based nonparametric test [13] to the same WTCCC case-control BD data. Moreover, a meta-analysis (including WTCCC, STEP-BD, Iceland and Scandinavia samples; *n* = 5547 BD cases and 20241 controls) [14] suggested association between rs420259 and BD (*P* =1.2E-5). However, such association was not further reported by GWAS in extended datasets ([15], see Craddock and Sklar for review [16]), which were based on the CAT_ADD test.

The rs420259 is located in an intron of PALB2 gene which is involved in tumor suppression. Interestingly, the DCTN5 gene is in the immediate vicinity of the PALB2 gene. DCTN5 is known to be involved in intracellular transport, and its knockdown in vitro leads to an abnormal hyper-activity and disrupted development of neural networks [17]. DCTN5 also interacts with DISC1 gene (Disrupted in schizophrenia 1), a gene associated with bipolar disorder in several studies [18].

## Conclusions

Overall, the GRM modeling framework is a user-friendly and powerful approach which allows testing for association with disease and for the underlying genetic model. This association test is easy and quick to apply and thus particularly appropriate for association studies of large panels of markers in simple and complex situations.

## Additional files

Additional file 1:
**Table S1.** and **Table S2.** reported the power of GRM and CAT tests to detect association for a *P*-value threshold of 1.0E-5 (Table S1) or 1.0E-7 (Table S2) using a sample size of 2000 cases/2000 controls. **Table S1.** GRM and CAT tests’ powers to detect association (*P*-value threshold ≤1.0E-5; *N* = 2000 cases/2000 controls). **Table S2**. GRM and CAT tests’ powers to detect association (*P*-value threshold ≤1.0E-7; *N* = 2000 cases/2000 controls) (ZIP 301 kb)
Additional file 2:
**Figure S1.** reported the proportion of replicates rejecting the additive model using a threshold *P*-value of 1.0E-5. **Figure S1.** Proportion of replicates rejecting the additive model (*P* = 0.01) among replicates showing significant association (*P*-value threshold =1.0E-5). (PDF 189 kb)
Additional file 3:
**Appendix S1.** and **Appendix S2.** reported shell and Pearl scripts which included PLINK commands [19]) used to analyze real dataset and to perform all simulations, computation of type I error and power estimations. (ZIP 33 kb)

## Abbreviations

BD: Bipolar disorder
CAT: Cochran-Armitage trend
CAT_ADD: Cochran-Armitage additive trend test
CAT_DOM: Cochran-Armitage dominant trend test
CAT_REC: Cochran-Armitage recessive trend test
DCTN5: Dynactin Subunit 5
Df: Degree of freedom
DISC1: Disrupted In Schizophrenia 1
GRM: General regression model
GWAS: Genome wide association studies
H0: Null hypothesis
HW: Hardy-Weinberg
MAF: Minor allele frequency
MAX: Maximum of the standardized optimal tests
MERT: Maximin efficiency robust test
NS: Non significant
OR: Odds-ratio
PALB2: Partner and localizer of BRCA2
QC: Quality control
S: Significant
SNP: Single-nucleotide polymorphism
WTCCC: Welcome trust case control cohort
