# Supplementary material for: Gain of power of the general regression model compared to Cochran-Armitage Trend tests: simulation study and application to bipolar disorder
Source: BMC Genet. 2017 Mar 10;18:24. doi: 10.1186/s12863-017-0486-6 (PMC5345257; doi:10.1186/s12863-017-0486-6)
Supplement: Additional file 1: — Table S1. and Table S2. reported the power of GRM and CAT tests to detect association for a P-value threshold of 1.0E-5 (Table S1) or 1.0E-7 (Table S2) using a sample size of 2000 cases/2000 controls. Table S1. GRM and CAT tests’ powers to detect association (P-value threshold ≤1.0E-5; N = 2000 cases/2000 controls). Table S2. GRM and CAT tests’ powers to detect association (P-value threshold ≤1.0E-7; N = 2000 cases/2000 controls) (ZIP 301 kb) [file 12863_2017_486_MOESM1_ESM.zip › GRM_Dizier_Table_S2.pdf]

		Simulated model : Additive				Simulated model : Dominant				Simulated model : Recessive			
		Tests :				Tests :				Tests :			
MAF	OR	CAT_DOM	CAT_REC	CAT_ADD	GRM	CAT_DOM	CAT_REC	CAT_ADD	GRM	CAT_DOM	CAT_REC	CAT_ADD	GRM
0.1	1.2	0	0	0	0	0	0	0	0	0	0	0	0
0.1	1.4	0.34	0	0.40	0.28	0.24	0	0.20	0.16	0	0	0	0
0.1	1.6	0.96	0.01	0.98	0.95	0.90	0	0.86	0.83	0	0	0	0
0.1	1.8	1	0.15	1	1	1	0	1	1	0	0	0	0
0.1	2	1	0.52	1	1	1	0	1	1	0	0	0	0
0.1	2.2	1	0.87	1	1	1	0	1	1	0	0.01	0	0
0.1	2.4	1	0.99	1	1	1	0	1	1	0	0.02	0	0
0.1	2.6	1	1	1	1	1	0	1	1	0	0.06	0	0
0.1	2.8	1	1	1	1	1	0	1	1	0	0.14	0	0.01
0.1	3	1	1	1	1	1	0.01	1	1	0	0.25	0	0.04
0.1	3.2	1	1	1	1	1	0.01	1	1	0	0.38	0	0.09
0.2	1.2	0.02	0	0.03	0.02	0.01	0	0.01	0	0	0	0	0
0.2	1.4	0.81	0.09	0.91	0.85	0.56	0	0.43	0.45	0	0	0	0
0.2	1.6	1	0.71	1	1	0.99	0	0.97	0.98	0	0.02	0	0.01
0.2	1.8	1	0.99	1	1	1	0	1	1	0	0.16	0	0.08
0.2	2	1	1	1	1	1	0.01	1	1	0	0.48	0.03	0.33
0.2	2.2	1	1	1	1	1	0.02	1	1	0	0.80	0.09	0.67
0.2	2.4	1	1	1	1	1	0.04	1	1	0	0.95	0.23	0.90
0.2	2.6	1	1	1	1	1	0.07	1	1	0	0.99	0.44	0.98
0.2	2.8	1	1	1	1	1	0.11	1	1	0.01	1	0.66	1
0.2	3	1	1	1	1	1	0.16	1	1	0.02	1	0.83	1
0.2	3.2	1	1	1	1	1	0.23	1	1	0.04	1	0.93	1
0.3	1.2	0.04	0.01	0.09	0.05	0.01	0	0	0.01	0	0	0	0
0.3	1.4	0.91	0.49	0.98	0.97	0.59	0	0.37	0.48	0	0.03	0	0.01
0.3	1.6	1	0.99	1	1	0.99	0	0.95	0.98	0	0.34	0.04	0.23
0.3	1.8	1	1	1	1	1	0	1	1	0	0.84	0.22	0.74
0.3	2	1	1	1	1	1	0.02	1	1	0	0.99	0.60	0.97
0.3	2.2	1	1	1	1	1	0.04	1	1	0.02	1	0.89	1
0.3	2.4	1	1	1	1	1	0.07	1	1	0.05	1	0.98	1
0.3	2.6	1	1	1	1	1	0.12	1	1	0.11	1	1	1
0.3	2.8	1	1	1	1	1	0.18	1	1	0.22	1	1	1
0.3	3	1	1	1	1	1	0.24	1	1	0.37	1	1	1
0.3	3.2	1	1	1	1	1	0.31	1	1	0.54	1	1	1
0.4	1.2	0.04	0.02	0.14	0.09	0.01	0	0	0	0	0	0	0
0.4	1.4	0.90	0.79	0.99	0.99	0.45	0	0.19	0.35	0	0.16	0.02	0.10
0.4	1.6	1	1	1	1	0.97	0	0.78	0.94	0	0.81	0.32	0.72
0.4	1.8	1	1	1	1	1	0	0.98	1	0.01	0.99	0.82	0.99
0.4	2	1	1	1	1	1	0.01	1	1	0.03	1	0.99	1
0.4	2.2	1	1	1	1	1	0.03	1	1	0.11	1	1	1
0.4	2.4	1	1	1	1	1	0.05	1	1	0.25	1	1	1
0.4	2.6	1	1	1	1	1	0.08	1	1	0.45	1	1	1
0.4	2.8	1	1	1	1	1	0.12	1	1	0.66	1	1	1
0.4	3	1	1	1	1	1	0.16	1	1	0.82	1	1	1
0.4	3.2	1	1	1	1	1	0.20	1	1	0.92	1	1	1
