# Supplementary material for: Gain of power of the general regression model compared to Cochran-Armitage Trend tests: simulation study and application to bipolar disorder
Source: BMC Genet. 2017 Mar 10;18:24. doi: 10.1186/s12863-017-0486-6 (PMC5345257; doi:10.1186/s12863-017-0486-6)
Supplement: Additional file 1: — Table S1. and Table S2. reported the power of GRM and CAT tests to detect association for a P-value threshold of 1.0E-5 (Table S1) or 1.0E-7 (Table S2) using a sample size of 2000 cases/2000 controls. Table S1. GRM and CAT tests’ powers to detect association (P-value threshold ≤1.0E-5; N = 2000 cases/2000 controls). Table S2. GRM and CAT tests’ powers to detect association (P-value threshold ≤1.0E-7; N = 2000 cases/2000 controls) (ZIP 301 kb) [file 12863_2017_486_MOESM1_ESM.zip › GRM_Dizier_Table_S1.pdf]

		Simulated model : Additive				Simulated model : Dominant				Simulated model : Recessive			
		Tests :				Tests :				Tests :			
MAF	OR	CAT_DOM	CAT_REC	CAT_ADD	GRM	CAT_DOM	CAT_REC	CAT_ADD	GRM	CAT_DOM	CAT_REC	CAT_ADD	GRM
0.1	1.2	0.03	0	0.04	0.02	0.02	0	0.02	0.01	0	0	0	0
0.1	1.4	0.69	0.01	0.75	0.63	0.58	0	0.53	0.46	0	0	0	0
0.1	1.6	1	0.13	1	1	0.99	0	0.98	0.97	0	0	0	0
0.1	1.8	1	0.5	1	1	1	0	1	1	0	0.01	0	0
0.1	2	1	0.86	1	1	1	0	1	1	0	0.03	0	0
0.1	2.2	1	0.98	1	1	1	0.01	1	1	0	0.09	0	0.02
0.1	2.4	1	1	1	1	1	0.02	1	1	0	0.18	0	0.05
0.1	2.6	1	1	1	1	1	0.04	1	1	0	0.32	0.01	0.12
0.1	2.8	1	1	1	1	1	0.06	1	1	0	0.47	0.02	0.22
0.1	3	1	1	1	1	1	0.09	1	1	0	0.63	0.03	0.37
0.1	3.2	1	1	1	1	1	0.12	1	1	0	0.77	0.05	0.52
0.2	1.2	0.13	0.01	0.18	0.12	0.07	0	0.05	0.04	0	0	0	0
0.2	1.4	0.96	0.35	0.99	0.97	0.86	0	0.77	0.78	0	0.02	0	0.01
0.2	1.6	1	0.93	1	1	1	0.01	1	1	0	0.15	0.01	0.08
0.2	1.8	1	1	1	1	1	0.02	1	1	0	0.48	0.05	0.34
0.2	2	1	1	1	1	1	0.06	1	1	0	0.82	0.15	0.7
0.2	2.2	1	1	1	1	1	0.12	1	1	0.01	0.96	0.34	0.92
0.2	2.4	1	1	1	1	1	0.2	1	1	0.02	1	0.58	0.99
0.2	2.6	1	1	1	1	1	0.29	1	1	0.04	1	0.78	1
0.2	2.8	1	1	1	1	1	0.39	1	1	0.07	1	0.91	1
0.2	3	1	1	1	1	1	0.49	1	1	0.12	1	0.97	1
0.2	3.2	1	1	1	1	1	0.57	1	1	0.19	1	0.99	1
0.3	1.2	0.21	0.05	0.34	0.24	0.08	0	0.05	0.05	0	0	0	0
0.3	1.4	0.99	0.82	1	1	0.87	0	0.72	0.8	0	0.16	0.02	0.1
0.3	1.6	1	1	1	1	1	0.01	0.99	1	0	0.7	0.19	0.58
0.3	1.8	1	1	1	1	1	0.05	1	1	0.01	0.97	0.56	0.94
0.3	2	1	1	1	1	1	0.11	1	1	0.04	1	0.88	1
0.3	2.2	1	1	1	1	1	0.19	1	1	0.11	1	0.98	1
0.3	2.4	1	1	1	1	1	0.29	1	1	0.22	1	1	1
0.3	2.6	1	1	1	1	1	0.4	1	1	0.38	1	1	1
0.3	2.8	1	1	1	1	1	0.5	1	1	0.56	1	1	1
0.3	3	1	1	1	1	1	0.59	1	1	0.72	1	1	1
0.3	3.2	1	1	1	1	1	0.66	1	1	0.84	1	1	1
0.4	1.2	0.21	0.12	0.43	0.32	0.06	0	0.02	0.03	0	0.02	0	0.01
0.4	1.4	0.99	0.96	1	1	0.79	0	0.51	0.7	0	0.47	0.15	0.36
0.4	1.6	1	1	1	1	1	0.01	0.96	0.99	0.01	0.96	0.68	0.93
0.4	1.8	1	1	1	1	1	0.04	1	1	0.06	1	0.97	1
0.4	2	1	1	1	1	1	0.09	1	1	0.18	1	1	1
0.4	2.2	1	1	1	1	1	0.15	1	1	0.37	1	1	1
0.4	2.4	1	1	1	1	1	0.23	1	1	0.6	1	1	1
0.4	2.6	1	1	1	1	1	0.31	1	1	0.79	1	1	1
0.4	2.8	1	1	1	1	1	0.39	1	1	0.91	1	1	1
0.4	3	1	1	1	1	1	0.47	1	1	0.97	1	1	1
0.4	3.2	1	1	1	1	1	0.54	1	1	0.99	1	1	1
