# Supplementary figures and images for: Gain of power of the general regression model compared to Cochran-Armitage Trend tests: simulation study and application to bipolar disorder
Source: BMC Genet. 2017 Mar 10;18:24. doi: 10.1186/s12863-017-0486-6 (PMC5345257; doi:10.1186/s12863-017-0486-6)

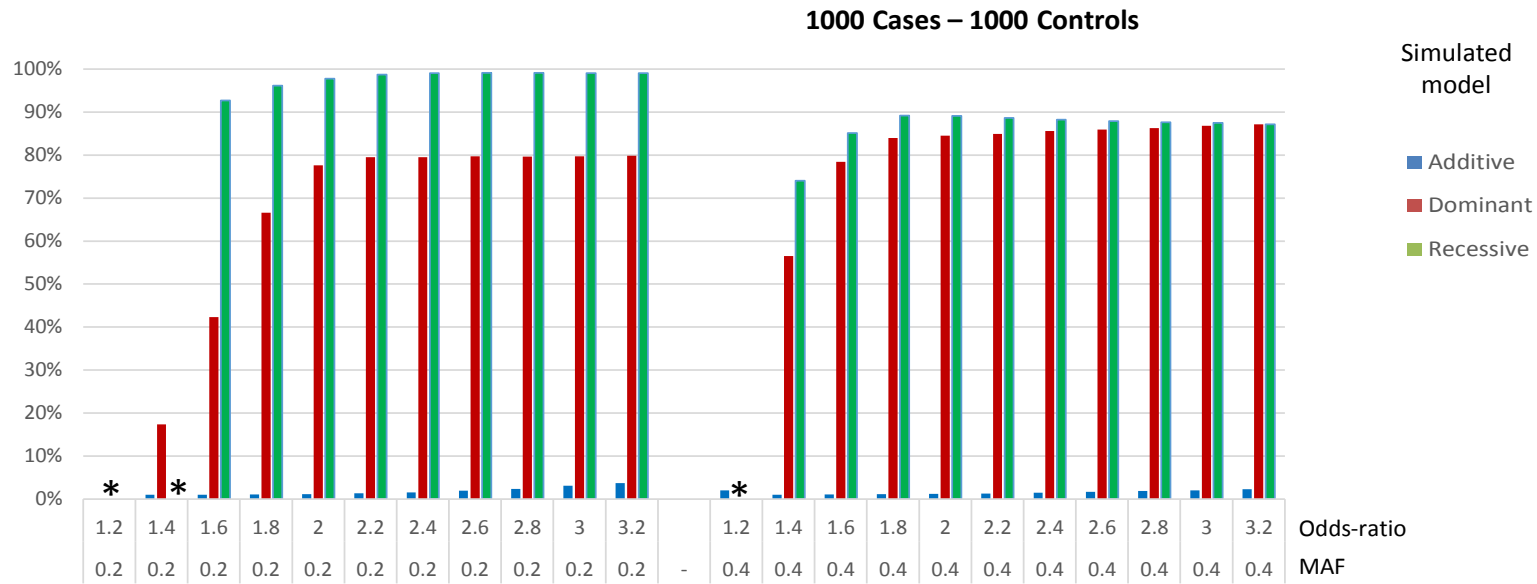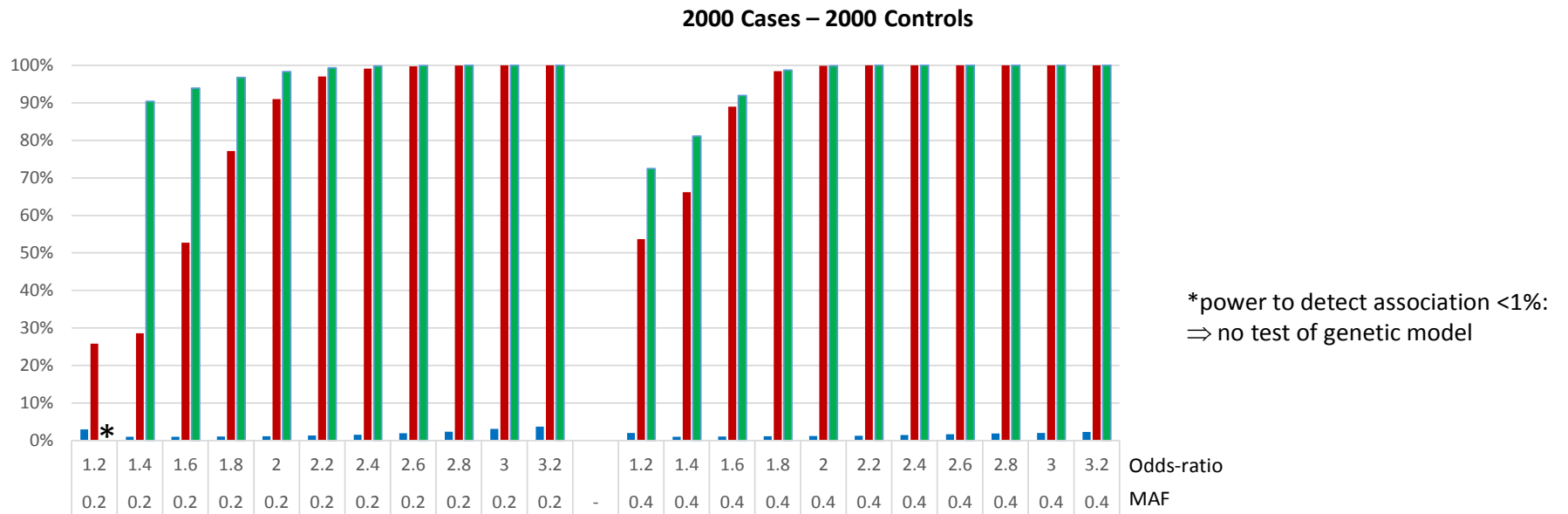

Supplement: Additional file 2: — Figure S1. reported the proportion of replicates rejecting the additive model using a threshold P-value of 1.0E-5. Figure S1. Proportion of replicates rejecting the additive model (P = 0.01) among replicates showing significant association (P-value threshold =1.0E-5). (PDF 189 kb) [file 12863_2017_486_MOESM2_ESM.pdf]
