# Supplementary material for: Gain of power of the general regression model compared to Cochran-Armitage Trend tests: simulation study and application to bipolar disorder
Source: BMC Genet. 2017 Mar 10;18:24. doi: 10.1186/s12863-017-0486-6 (PMC5345257; doi:10.1186/s12863-017-0486-6)
Supplement: Additional file 3: — Appendix S1. and Appendix S2. reported shell and Pearl scripts which included PLINK commands [19]) used to analyze real dataset and to perform all simulations, computation of type I error and power estimations. (ZIP 33 kb) [file 12863_2017_486_MOESM3_ESM.zip › GRM_Dizier_AppendixS2.docx]

**S2 APPENDIX: Scripts for Simulation studies**

**S2.1- Script used to generate 200 000 replicates for the 396 genetic models for each sample sizes (N=1000 and N=2000 cases and controls samples) and perform association analysis**

#!/bin/bash

for model in rec dom add **# Genetic underlying model**

do for prevalence in 0.01 0.05 0.10 **# Disease prevalences**

do mal=`echo $prevalence | sed 's/0\.//' `

for snp in 0.1 0.2 0.3 0.4 **# SNP’s allele frequencies**

do

snpp=`echo $snp | sed 's/\.//' `

dirname=`echo $model"_"$mal"_"$snpp`

dirres=`echo $model"_"$mal"_"$snpp"_res"`

if [ -d $dirname ]

then

cd $diname

else

mkdir $dirname

mkdir $dirres

cd $dirname

fi

for or in 1.0 1.2 1.4 1.6 1.8 2.0 2.2 2.4 2.6 2.8 3.0 3.2

**# Odds-ratio. 1.0 for robustness estimation, others for power comparison**

do

orr=`echo $or | sed 's/\.//' `

filename=`echo $dirname"_"$orr`

echo $filename

# create sim file

if [ "$model" = "dom" ]

then

echo "200000" $model $snp $snp $or $or > $model.sim **# Number of replicates (model DOM)**

fi

if [ "$model" = "add" ]

then

echo "200000" $model $snp $snp $or "mult" > $model.sim **# Number of replicates (mod. ADD)**

fi

if [ "$model" = "rec" ]

then

echo "200000" $model $snp $snp "1" $or > $model.sim **# Number of replicates (model REC)**

fi

plink --simulate $model.sim --simulate-ncases 1000 --simulate-ncontrols 1000 --simulate-prevalence $prevalence --make-bed --out $filename --noweb > /dev/null

**# Number of cases and controls 1000 or 2000.**

plink --file $filename --model --reference-allele ./**MARK.txt** --cell 2 --out $filename --noweb > /dev/null

**# Association Analysis using CAT_ tests**

plink --file $filename --logistic --genotypic --reference-allele ../MARK.txt --out $filename --noweb > /dev/null

**# Association Analysis using GRM test**

mv $filename.model ../$dirres/$filename.model_1000

mv $filename.assoc.logistic ../$dirres/$filename.assoc.logistic_1000

gzip ../$dirres/$filename.model_1000

gzip ../$dirres/$filename.assoc.logistic_1000

done

cd ..

tar czvf $dirname.tar.gz $dirname > /dev/null

rm -r $dirname

done

done

done

**./MARK.txt (definition of the reference allele in PLINK)**

marq_0 d

marq_1 d

marq_2 d

marq_3 d

…

marq_n d

**# Script used to generate summary tables**

#!/bin/bash

for model in rec dom add **# Genetic underlying models**

do

if [ -s Power.$model ]

then

rm Power.$model

fi

echo "H0;model;maladie;snp;or;na_dom;np_5dom;np_7dom;na_rec;np_5rec;np_7rec;na_add;np_5add;np_7add;na_GEN_ADD; np_5GEN_ADD; np_7GEN_ADD; na_GEN_DOMDEV;np_5GEN_DOMDEV; np75GEN_DOMDEV;na_GENERAL; np_5GENERAL;np_7GENERAL" >> Power1000_01_add.$model

**# Header of the summary table**

for prevalence in 0.01 0.05 0.10 **# Disease prevalence**

do mal=`echo $prevalence | sed 's/0\.//' `

for snp in 0.1 0.2 0.3 0.4 **# SNP’s allele frequencies**

do

snpp=`echo $snp | sed 's/\.//' `

dirname=`echo $model"_"$mal"_"$snpp`

dirres=`echo $model"_"$mal"_"$snpp"_res"`

for or in 1.2 1.4 1.6 1.8 2.0 2.2 2.4 2.6 2.8 3.0 3.2 **# Odds-ratio**

do

orr=`echo $or | sed 's/\.//' `

filename=`echo $dirname"_"$orr`

echo $filename

gunzip $filename.model.gz

na_dom=`grep "DOM" $filename.model | grep "NA" | wc -l | awk '{print$1}' `

na_rec=`grep "REC" $filename.model | grep "NA" | wc -l | awk '{print$1}' `

na_add=`grep "TREND" $filename.model | grep "NA" | wc -l | awk '{print$1}' `

**S2.2- Power estimation (number of p-values < threshold divided by the number of replicates)**

**# Power for CAT_ tests**

**# For CAT_DOM test**

grep "DOM" $filename.model | grep -v "NA" | awk '{print$10}' > tmp1

if [ -s tmp1 ]

then

np_5dom=`awk '$1 <= 0.00001{print}' tmp1 | wc -l | awk '{print$1}' `

**# for a threshold of 10^-5^**

np_7dom=`awk '$1 <= 0.0000001{print}' tmp1 | wc -l | awk '{print$1}' `

**# for a threshold of 10^-7^**

rm tmp1

fi

**# For CAT_REC test**

grep "REC" $filename.model | grep -v "NA" | awk '{print$10}' > tmp2

if [ -s tmp2 ]

then

np_5rec=`awk '$1 <= 0.00001{print}' tmp2 | wc -l | awk '{print$1}' `

**# for a threshold of 10^-5^**

np_7rec=`awk '$1 <= 0.0000001{print}' tmp2 | wc -l | awk '{print$1}' `

**# for a threshold of 10^-7^**

rm tmp2

fi

**# For CAT_ADD test**

grep "TREND" $filename.model | grep -v "NA" | awk '{print$10}' > tmp3

if [ -s tmp3 ]

then

np_5trend=`awk '$1 <= 0.00001{print}' tmp3 | wc -l | awk '{print$1}' `

**# for a threshold of 10^-5^**

np_7trend=`awk '$1 <= 0.0000001{print}' tmp3 | wc -l | awk '{print$1}' `

**# for a threshold of 10^-7^**

rm tmp3

fi

**# Power estimation for GRM**

gunzip $filename.assoc.logistic.gz

na_GEN_ADD=`grep "ADD" $filename.assoc.logistic | wc -l | awk '{print$1}' `

na_GEN_DOMDEV=`grep "DOMDEV" $filename.assoc.logistic | wc -l | awk '{print$1}' `

na_GENERAL=`grep "GENO_2DF" $filename.assoc.logistic | wc -l | awk '{print$1}' `

**# For ADD parameter**

grep "ADD" $filename.assoc.logistic | grep -v "NA" | awk '{print$9}' > tmp6

if [ -s tmp6 ]

then

np_5GEN_ADD=`awk '$1 <= 0.00001{print}' tmp6 | wc -l | awk '{print$1}' `

**# for a threshold of 10^-5^**

np_7GEN_ADD=`awk '$1 <= 0.0000001{print}' tmp6 | wc -l | awk '{print$1}' `

**# for a threshold of 10^-7^**

fi

grep "ADD" $filename.assoc.logistic | grep -v "NA" | awk '{print$7}' > tmp7

if [ -s tmp7 ]

then # Si tmp7 non vide

grep "ADD" $filename.assoc.logistic | awk '{print $7, $9}' > toto

awk '$2 <= 0.01{print $1, $2}' toto > toto2

na_ORADD= wc -l toto2 | awk '{print$1}'

fi

rm tmp6 tmp7 toto toto2

**# For DOMDEV parameter**

grep "DOMDEV" $filename.assoc.logistic | grep -v "NA" | awk '{print$9}' > tmp8

if [ -s tmp8 ]

then

np_5GEN_DOMDEV=`awk '$1 <= 0.00001{print}' tmp8 | wc -l | awk '{print$1}' `

**# for a threshold of 10^-5^**

np_7GEN_DOMDEV=`awk '$1 <= 0.0000001{print}' tmp8 | wc -l | awk '{print$1}' `

**# for a threshold of 10^-7^**

fi

grep "DOMDEV" $filename.assoc.logistic | grep -v "NA" | awk '{print$7}' > tmp9

if [ -s tmp9 ]

then # calcul de la moyenne des OR quand p<0.05

grep "DOMDEV" $filename.assoc.logistic | awk '{print $7, $9}' > toto

awk '$2 <= 0.05{print $1, $2}' toto > toto2

na_ORDOMDEV= wc -l toto2 | awk '{print$1}'

fi

rm tmp8 tmp9 toto toto2

**# For GRM test**

grep "GENO_2DF" $filename.assoc.logistic | awk '{print$9}' > tmp10

if [ -s tmp10 ]

then

np_5GENERAL=`awk '$1 <= 0.00001{print}' tmp10 | wc -l | awk '{print$1}' `

**# for a threshold of 10^-5^**

np_7GENERAL=`awk '$1 <= 0.0000001{print}' tmp10 | wc -l | awk '{print$1}' `

**# for a threshold of 10^-7^**

echo $np5GENERAL

rm tmp10

fi

echo "H0;"$model";"$maladie";"$snp";"$or";"$na_dom";"$np_5dom";"$np_7dom";"$na_rec";"$np_5rec";"$np_7rec";"$na_addd";"$np_5add";$np_7add";"$na_GEN_ADD";"$np5GEN_ADD";"$np1GEN_ADD";"$np_5GEN_ADD";"$na_GEN_DOMDEV";"$np5GEN_DOMDEV";"$np1GEN_DOMDEV";"$np_5GEN_DOMDEV";"$na_GENERAL";"$np_5GENERAL";"$np_7GENERAL";" >> Power1000$model

gzip $filename.model

gzip $filename.assoc.logistic

done

done

done

done

**S2.3- Script to Test for genetic model**

#!/bin/bash

path="/works/efige/N1000_RESULTS/RES_05" **# Path for association test results**

for model in rec dom add

do

if [ -s resultat.$model ]

then

rm resultat.$model

fi

for prevalence in 0.01 0.05 0.10

do

mal=`echo $prevalence | sed 's/0\.//' `

for snp in 0.1 0.2 0.3 0.4

do

snpp=`echo $snp | sed 's/\.//' `

dirname=`echo $model"_"$mal"_"$snpp`

dirres=`echo $model"_"$mal"_"$snpp"_res"`

for or in 1.2 1.4 1.6 1.8 2 2.2 2.4 2.6 2.8 3 3.2

do

orr=`echo $or | sed 's/\.//' `

filename=`echo $dirname"_"$orr`

echo $filename

gunzip $path"/"$filename.assoc.logistic.gz

echo $filename >> name

**./Model.pl** $filename.assoc.logistic >> count **# Pearl script described bellow**

paste name count >> Count_Models_05.$model

gzip $path"/"$filename.assoc.logistic

done

rm name count

done

done

done

**# Model.pl**

#!/usr/bin/perl

use strict; **# turn on compiler restrictions**

my $entete;

my $ADD_OR;

my $ADD_STAT;

my $ADD_P;

my $ADD_DEBUT;

my $DOM_OR;

my $DOM_STAT;

my $DOM_P;

my $DOM_DEBUT;

my $GENO_OR;

my $GENO_STAT;

my $GENO_P;

my $GENO_DEBUT;

my $BETA_ADD;

my $BETA_DOMDEV;

my $SD_BETA_ADD;

my $IC_min;

my $IC_max;

my $IC_MOINSBETA_min;

my $IC_MOINSBETA_max;

my $count_DOM ;

my $count_REC ;

my $count_Gal;

my $count_GalNS;

my $count_DomDev;

my @line;

my $i;

$i=0;

$count_DOM =0;

$count_REC =0;

$count_Gal=0;

$count_GalNS=0;

$count_DomDev=0;

$entete=<>;

while (<>) {

#print $_;

#Split line in columns @line = split(/ +/, $_); chomp @line;

#print @line;

if ($line[5]=~/ADD/) {

$ADD_DEBUT=$line[1]." ".$line[2]." ".$line[3];

$ADD_OR=$line[7];

$ADD_STAT=$line[8];

$ADD_P=$line[9];

#print "ADD : ", $ADD_DEBUT, " | ", $ADD_OR, " ",$ADD_STAT," ", $ADD_P, "\n";

}

if ($line[5]=~/DOMDEV/) {

$DOM_DEBUT=$line[1]." ".$line[2]." ".$line[3];

$DOM_OR=$line[7];

$DOM_STAT=$line[8];

$DOM_P=$line[9];

# print "DOM : ", $DOM_DEBUT, " | ", $DOM_OR, " ",$DOM_STAT," ", $DOM_P, "\n";

}

if ($line[5]=~/GENO_2DF/) {

$GENO_DEBUT=$line[1]." ".$line[2]." ".$line[3];

$GENO_OR=$line[7];

$GENO_STAT=$line[8];

$GENO_P=$line[9];

#print "GENO : ", $GENO_DEBUT," | ", $GENO_OR, " ", $GENO_STAT," ", $GENO_P, "\n";

}

if ("$ADD_DEBUT" eq "$DOM_DEBUT" and "$ADD_DEBUT" eq "$GENO_DEBUT") {

#print "tout va bien\n";

if ($GENO_P <= 0.05) {$count_Gal ++;}

if ($GENO_P > 0.05) {$count_GalNS ++;}

if ($GENO_P <= 0.05 && $DOM_P <= 0.05) {$count_DomDev ++;}

if ($GENO_P <= 0.05 && $DOM_P <=0.05 && $ADD_OR !=0 && $DOM_OR !=0) {

$BETA_ADD=log($ADD_OR);

$BETA_DOMDEV=log($DOM_OR);

$SD_BETA_ADD=($BETA_ADD/$ADD_STAT);

$IC_min=($BETA_ADD -(**2.58***$SD_BETA_ADD));

$IC_max=($BETA_ADD +(**2.58***$SD_BETA_ADD));

$IC_MOINSBETA_min = (-$BETA_ADD -(**2.58***$SD_BETA_ADD));

$IC_MOINSBETA_max = (-$BETA_ADD +(**2.58***$SD_BETA_ADD));

**# Must be change according to the p-value threshold for significance: 1.96 for 5%, 2.58 for 1%**

if ($BETA_DOMDEV >= $IC_min && $BETA_DOMDEV <= $IC_max)

{$count_DOM ++;}

else {if ($BETA_DOMDEV >= $IC_MOINSBETA_min && $BETA_DOMDEV <= $IC_MOINSBETA_max)

{$count_REC ++;}

}

#printf ( "$ADD_DEBUT;$ADD_OR;$ADD_STAT;$ADD_P;$DOM_OR;$DOM_P;$GENO_P;$BETA_ADD;$SD_BETA_ADD;$BETA_DOMDEV;$IC_min;$IC_max;$IC_MOINSBETA_min;$IC_MOINSBETA_max;$count_DOM;$count_REC\n");

}

# print "BETA_ADD= $BETA_ADD\n";

# print "SD BETA_ADD= $SD_BETA_ADD\n";

# print "BETA_DOMDEV= $BETA_DOMDEV\n";

# print "IC_BETA_ADD_min= $IC_min\n";

# print "IC_BETA_ADD_max= $IC_max\n";

# print "COUNT DOM= $count_DOM\n";

# print "COUNT_REC= $count_REC\n";

}

}

printf ("$count_DOM;$count_REC;$count_Gal;$count_GalNS;$count_DomDev\n")
