# Supplementary material for: Gain of power of the general regression model compared to Cochran-Armitage Trend tests: simulation study and application to bipolar disorder
Source: BMC Genet. 2017 Mar 10;18:24. doi: 10.1186/s12863-017-0486-6 (PMC5345257; doi:10.1186/s12863-017-0486-6)
Supplement: Additional file 3: — Appendix S1. and Appendix S2. reported shell and Pearl scripts which included PLINK commands [19]) used to analyze real dataset and to perform all simulations, computation of type I error and power estimations. (ZIP 33 kb) [file 12863_2017_486_MOESM3_ESM.zip › GRM_Dizier_AppendixS1.docx]

**APPENDIX S1: Script to analyze real dataset**

**S11- Script to Test for association**

plink --file filename --logistic --genotypic --reference-allele ../MARK.txt --out filename

**S12- Script to Test for genetic model**

Model.pl filename.assoc.logistic >> Count_Models_05.model **# Pearl script described bellow**

**# Model.pl**

#!/usr/bin/perl

use strict; **# turn on compiler restrictions**

my $entete;

my $ADD_OR;

my $ADD_STAT;

my $ADD_P;

my $ADD_DEBUT;

my $DOM_OR;

my $DOM_STAT;

my $DOM_P;

my $DOM_DEBUT;

my $GENO_OR;

my $GENO_STAT;

my $GENO_P;

my $GENO_DEBUT;

my $BETA_ADD;

my $BETA_DOMDEV;

my $SD_BETA_ADD;

my $IC_min;

my $IC_max;

my $IC_MOINSBETA_min;

my $IC_MOINSBETA_max;

my $count_DOM ;

my $count_REC ;

my $count_Gal;

my $count_GalNS;

my $count_DomDev;

my @line;

my $i;

$i=0;

$count_DOM =0;

$count_REC =0;

$count_Gal=0;

$count_GalNS=0;

$count_DomDev=0;

$entete=<>;

while (<>) {

#print $_;

#Split line in columns @line = split(/ +/, $_); chomp @line;

#print @line;

if ($line[5]=~/ADD/) {

$ADD_DEBUT=$line[1]." ".$line[2]." ".$line[3];

$ADD_OR=$line[7];

$ADD_STAT=$line[8];

$ADD_P=$line[9];

#print "ADD : ", $ADD_DEBUT, " | ", $ADD_OR, " ",$ADD_STAT," ", $ADD_P, "\n";

}

if ($line[5]=~/DOMDEV/) {

$DOM_DEBUT=$line[1]." ".$line[2]." ".$line[3];

$DOM_OR=$line[7];

$DOM_STAT=$line[8];

$DOM_P=$line[9];

# print "DOM : ", $DOM_DEBUT, " | ", $DOM_OR, " ",$DOM_STAT," ", $DOM_P, "\n";

}

if ($line[5]=~/GENO_2DF/) {

$GENO_DEBUT=$line[1]." ".$line[2]." ".$line[3];

$GENO_OR=$line[7];

$GENO_STAT=$line[8];

$GENO_P=$line[9];

#print "GENO : ", $GENO_DEBUT," | ", $GENO_OR, " ", $GENO_STAT," ", $GENO_P, "\n";

}

if ("$ADD_DEBUT" eq "$DOM_DEBUT" and "$ADD_DEBUT" eq "$GENO_DEBUT") {

#print "tout va bien\n";

if ($GENO_P <= 0.05) {$count_Gal ++;}

if ($GENO_P > 0.05) {$count_GalNS ++;}

if ($GENO_P <= 0.05 && $DOM_P <= 0.05) {$count_DomDev ++;}

if ($GENO_P <= 0.05 && $DOM_P <=0.05 && $ADD_OR !=0 && $DOM_OR !=0) {

$BETA_ADD=log($ADD_OR);

$BETA_DOMDEV=log($DOM_OR);

$SD_BETA_ADD=($BETA_ADD/$ADD_STAT);

$IC_min=($BETA_ADD -(**2.58***$SD_BETA_ADD));

$IC_max=($BETA_ADD +(**2.58***$SD_BETA_ADD));

$IC_MOINSBETA_min = (-$BETA_ADD -(**2.58***$SD_BETA_ADD));

$IC_MOINSBETA_max = (-$BETA_ADD +(**2.58***$SD_BETA_ADD));

**# Must be change according to the p-value threshold for significance: 1.96 for 5%, 2.58 for 1%**

if ($BETA_DOMDEV >= $IC_min && $BETA_DOMDEV <= $IC_max)

{$count_DOM ++;}

else {if ($BETA_DOMDEV >= $IC_MOINSBETA_min && $BETA_DOMDEV <= $IC_MOINSBETA_max)

{$count_REC ++;}

}

#printf ( "$ADD_DEBUT;$ADD_OR;$ADD_STAT;$ADD_P;$DOM_OR;$DOM_P;$GENO_P;$BETA_ADD;$SD_BETA_ADD;$BETA_DOMDEV;$IC_min;$IC_max;$IC_MOINSBETA_min;$IC_MOINSBETA_max;$count_DOM;$count_REC\n");

}

# print "BETA_ADD= $BETA_ADD\n";

# print "SD BETA_ADD= $SD_BETA_ADD\n";

# print "BETA_DOMDEV= $BETA_DOMDEV\n";

# print "IC_BETA_ADD_min= $IC_min\n";

# print "IC_BETA_ADD_max= $IC_max\n";

# print "COUNT DOM= $count_DOM\n";

# print "COUNT_REC= $count_REC\n";

}

}

printf ("$count_DOM;$count_REC;$count_Gal;$count_GalNS;$count_DomDev\n")
